# Cerebrospinal Fluid TNF-α and Orexin in Patients With Parkinson's Disease and Rapid Eye Movement Sleep Behavior Disorder

**DOI:** 10.3389/fneur.2022.826013

**Published:** 2022-02-18

**Authors:** Yuan Yuan, Yimeng Zhang, Yueyang Cheng, Yue Hou, Zhaoyang Huang, Jinghong Ma, Ning Li, Shuqin Zhan

**Affiliations:** ^1^Department of Neurology, Xuanwu Hospital, Capital Medical University, Beijing, China; ^2^Beijing Key Laboratory of Neuromodulation, Beijing, China; ^3^Beijing Institute of Brain Disorders, Collaborative Innovation Center for Brain Disorders, Capital Medical University, Beijing, China

**Keywords:** rapid eye movement sleep behavior disorder, Parkinson's disease, cerebrospinal fluid, TNF-α, orexin

## Abstract

**Background:**

Parkinson's disease (PD) pathological changes begin before motor symptoms appear. Rapid eye movement sleep behavior disorder (RBD) has the highest specificity and predictive value of any marker of prodromal PD. Tumor necrosis factor α (TNF-α) plays a part in the pathology of PD and disease conversion in isolated RBD (iRBD). TNF can also directly impair the hypocretin system in mice *in vivo*. As a result, we intend to investigate the effect of TNF-α on orexin levels in PD patients with RBD.

**Method:**

Participants were recruited from the Department of Neurology of Xuanwu Hospital, Capital Medical University to engage in assessments on motor symptoms, sleep, cognition, etc. Then we collected blood and cerebrospinal fluid of all patients and 10 controls' cerebrospinal fluid. The levels of TNF-α in the serum and cerebrospinal fluid, as well as the level of orexin in the cerebrospinal fluid, were measured in the patients.

**Results:**

The difference in TNF- levels in cerebrospinal fluid and serum between the three groups were not statistically significant. The levels of orexin in the three groups were not significantly lower than in the control group. UPDRS-III scores were significantly higher in the PD+RBD and PD-RBD groups than in the iRBD group. There was no statistically significant difference in H-Y stages, PSQI, or ESS scores between the PD+RBD and PD-RBD groups.

**Conclusion:**

Our findings suggest that TNF-α may not have a significant effect on the orexinergic system in patients with Parkinson's disease and iRBD. As a result, it is necessary to investigate the changes in TNF-α and orexin levels in different disease stages and to enlarge the sample size to determine whether TNF-α affects the function of the orexin system, which may be related to the occurrence of RBD and disease progression in Parkinson's disease.

## Introduction

Parkinson's disease (PD) is a common neurodegenerative disease characterized clinically by resting tremor, bradykinesia, rigidity, and postural balance disorder. The presence of fibrillar aggregates, known as Lewy bodies (LBs), in which α-synuclein is a major constituent, is a histopathological hallmark of Parkinson's disease (1). With the in-depth study of the disease, non-motor symptoms (NMS) have received increasing attention and have become a new research hotspot in Parkinson's disease. Hyposmia, autonomic dysfunction, anxiety and depression, cognitive impairment, and sleep disturbances are a few examples.

Rapid eye movement (REM) sleep behavior disorder (RBD) is a parasomnia characterized by loss of muscle atonia with abnormal dream-enacting behavior during REM sleep (2), which can result in injuries to these individuals and their bed partners. Idiopathic or isolated RBD (iRBD) is defined as RBD in the absence of any relevant neurological disorder or other precipitating factors (2). According to a longitudinal study, the majority of patients are eventually diagnosed with synucleinopathies such as Parkinson's disease (PD), dementia with Lewy bodies (DLB), and multiple system atrophy (MSA) (3). Multicenter prospective cohort studies have shown that over 60% of iRBD developed overt α-synucleinopathies in a decade or more and the phenoconversion rate was 6.25% per year (4). A meta-analysis confirmed a risk of more than 90% at 14 years (5). Furthermore, RBD confirmed by polysomnography (PSG) has by far the highest specificity and predictive value of any prodromal PD marker (6). According to Braak, α-synuclein deposition begins in the anterior olfactory nucleus, the dorsal motor nucleus of the vagus (7), implying that the pathological changes of PD begin before motor symptoms manifest. As a result, the clinical diagnosis for Parkinson's disease treatment may be delayed. For this reason, identifying reliable biomarkers is critical.

Orexins, also known as hypocretins, are neuropeptides produced by the hypothalamus and play a part in metabolism, feeding, reward, addiction, and sleep-wake control (8). According to the LU-SAPER model (9), orexin may indirectly participate in the innervation of spinal motor neurons by the sublaterodorsal tegmental nucleus (SLD) *via* fiber projections to the lateral pontine tegmentum (LPT), influencing REM atonia during sleep.

TNF-α, a potent pro-inflammatory cytokine, is recognized as an important mediator of neuroinflammation in the brain (10). TNF-α produced in large by activated microglia contributes to neuroinflammatory processes in a variety of neurological disorders (11). TNF-α has been shown to play a role in the pathology of Parkinson's disease (12) and disease conversion in iRBD (13). According to a study, TNF can impair the hypocretin system directly *in vivo* in mice. The research also suggests that repeated TNF challenge induces RBD-like behavior and sleep dysfunction in mice, as well as a decrease in learning, cognition, and memory (14).

As a result, we wonder if there are any links between the levels of TNF-α and orexin in cerebrospinal fluid (CSF) in patients with Parkinson's disease and rapid eye movement sleep behavior disorder. We recruited patients to investigate the above-mentioned correlations. TNF-α and orexin levels in cerebrospinal fluid and serum TNF-α were measured and compared.

## Method

### Participants

Patients with Parkinson's disease and iRBD were recruited for this study from the Department of Neurology of Xuanwu Hospital, Capital Medical University. To assess subjects on motor symptoms, sleep, cognition and emotion, we used Unified Parkinson's Disease Rating Scales part III (UPDRS-III), Hoehn-Yahr (H-Y) stage, Pittsburgh sleep quality index (PSQI), Epworth sleepiness scale (ESS), REM Sleep Behavior Disorder Questionnaire-Hong Kong (RBDQ-HK), Cognitive impairment uses Montreal Cognitive Assessment (MoCA), Hamilton rating scale for anxiety (HAMA) and Hamilton rating scale for depression (HAMD). Patients were classified in iRBD, PD with RBD (PD+RBD), PD without RBD (PD–RBD) groups. The following were the inclusion criteria for Parkinson's disease patients: (1) diagnosis refer to MDS clinical diagnostic criteria for Parkinson's disease (15), (2) age range of 40 to 80 years. The diagnosis of RBD refers to the International Classification of Sleep Disorders, Third Version (ICSD-3). Exclusion criteria were as follows: (1) those with parkinsonism from a cause other than Parkinson's disease, (2) those with a history of head injury, brain tumor, encephalitis, stroke; and abnormal electroencephalography (EEG) suggesting epilepsy, (3) those who take sleeping pills or antipsychotics, use sedative-hypnotic drugs or alcohol to help sleep and are unable to withdraw, (4) those who are unable to complete overnight PSG. The following criteria were used to select 10 age-matched control subjects: no neurodegenerative diseases, no sleep disorders, and no epilepsy. The overnight video-polysomnograph examination was performed and analyzed for 145 patients.

### Clinical Assessment

Patients were subjected to a thorough neurological examination, which included the Unified Parkinson's Disease Rating Scale part III (UPDRS III) and the Hoehn-Yahr (H-Y) stage for motor symptoms. The REM Sleep Behavior Disorder Questionnaire-Hong Kong (RBDQ-HK) was used to assess RBD. Daytime sleepiness and sleep quality were assessed using the Epworth sleepiness scale (ESS) and the Pittsburgh sleep quality index (PSQI). Cognitive impairment was evaluated using the Montreal Cognitive Assessment (MoCA). To quantify anxiety and depressive symptom severity, the Hamilton rating scale for anxiety (HAMA) and Hamilton rating scale for depression (HAMD) were used.

### Video-Polysomnograph

The Compumedics E-series polysomnography monitoring system, manufactured in Australia by Compumedics, was used to monitor patients' sleep throughout the night. The monitoring content includes EEG (electrodes installed in accordance with the international 10–20 system, respectively F3, F4, C3, C4, O1-A2, O2-A1), electrooculogram, chin, and both lower limbs EMG, mouth Nasal airflow (pressure sensing and thermal sensing), chest and abdomen breathing, electrocardiogram, and blood oxygen saturation.

### CSF and Serum Samples Collection and Analysis

CSF was collected from 20 patients *via* lumbar puncture standardized procedures and placed in siliconized polypropylene tubes. The samples were then centrifuged for 10 min at 4°C within 30 min of blood collection. After centrifugation, the extracted samples were stored at −80°C. The level of orexin was measured by radioimmunoassay. TNF-α level was measured by chemiluminescent immunoassay. TNF-α was evaluated using the IMMULITE/IMMULITE 1000 TNF-α kits (Siemens Healthcare Diagnostics, Llanberis, UK) according to manufacturer instructions. The average intra- and inter-assay coefficients of variation were 3.5% and 6.5%, respectively.

### Statistical Analysis

We used mean, standard deviation, median, range, and quartile to present data. The SPSS version 25 software package was used to perform all statistical analyses. One-way ANOVA was used to analyze data with a normal distribution. We used the Kruskal Wallis H test for non-normal distribution data. The chi-square test was used to analyze data for categorical variables. Pearson correlation analysis was used to assess the statistical relationship between TNF-α and orexin levels. A *p*-value of <0.05 was considered statistically significant.

## Results

### Demographics and Clinical Assessments

According to the inclusion criteria listed above, a total of 145 patients were studied, including 38 PD patients with RBD, 55 PD patients without RBD, and 52 iRBD patients. Clinical data were compared between the PD+RBD, PD-RBD, and iRBD groups (motor symptoms, non-motor symptoms: cognition, emotion, and sleep). There was no significant difference in age or gender ratio among the included patients. UPDRS-III scores were significantly higher in the PD+RBD and PD-RBD groups than in the iRBD group [22 (14.89, 34.50) vs. 17 (15.45, 26.50) vs. 1 (0, 2.25), *P* < 0.001]. There was no remarkable difference in H-Y stages, PSQI, ESS scores between PD+RBD and PD-RBD groups. However, RBDQ-HK scores in the iRBD group were significantly higher than those in the other groups with statistical significance (22.38 ± 18.74 vs. 15.66 ± 14.28 vs. 34.07 ± 19.16, *P* < 0.001). Although there was no statistically significant difference, the MoCA score in the PD+RBD group was lower than the PD-RBD group (21.01 ± 4.28 vs. 21.77 ± 4.38). The PD+RBD group's HAMA score was significantly higher than the iRBD group's (18.17 ± 5.82 vs. 14.92 ± 6.76, *P* = 0.004). Nonetheless, there was no discernible difference in HAMD scores between the three groups. Table 1 shows the demographics and clinical scores of study participants, as well as their comparison.

**Table 1:** Demographics and clinical tests including Unified Parkinson's Disease Rating Scale part III (UPDRS-III) and Hoehn-Yahr (H-Y) stage scores, Pittsburgh sleep quality index (PSQI), Epworth sleepiness scale (ESS), REM Sleep Behavior Disorder Questionnaire-Hong Kong (RBDQ-HK), Montreal Cognitive Assessment (MoCA), Hamilton rating scale for anxiety (HAMA), Hamilton rating scale for depression (HAMD) in Parkinson's disease (PD), isolated rapid eye movement sleep behavior disorder (iRBD) and PD with RBD subjects.

	**PD+RBD**		**PD-RBD**		**iRBD**		***P*-value**
	***N***		***N***		***N***		
Age (mean ± SD), years	38	64.35 ± 9.83	55	59.94 ± 9.17	52	63.33 ± 8.88	0.053
Gender (Male number/%)	38	22.00/57.89%	55	29.00/52.73%	52	33.00/63.46%	0.532
UPDRS-III [M, (P25, P75)]	9	22.00 (14.89, 34.50)	5	17.00 (15.45, 26.50)	10	1.00 (0, 2.25)	<0.001^b^^c^
H-Y stage (mean, range)	36	2.06, 1–4	48	2.03, 1–3	-	-	-
PSQI [M, (P25, P75)]	9	5.00 (4.50, 7.86)	5	8.00 (5.86, 16.50)	10	6.00 (2.75, 6.72)	0.115
ESS (mean ± SD)	37	4.50 ± 3.55	43	5.00 ± 4.24	48	4.88 ± 4.29	0.854
RBDQ-HK (mean ± SD)	37	22.38 ± 18.74	43	15.66 ± 14.28	48	34.07 ± 19.16	<0.001^a^^c^
MoCA (mean ± SD)	21	21.01 ± 4.28	21	21.77 ± 4.38	20	23.87 ± 3.11	0.067
HAMA (mean ± SD)	12	18.17 ± 5.82	6	14.92 ± 6.76	19	12.24 ± 6.00	0.040^b^
HAMD (mean ± SD)	12	13.92 ± 3.98	6	14.08 ± 4.59	19	8.92 ± 8.87	0.106
TIB [M, (P25, P75)], min		500.50 (518.00, 578.50)		545.75 (521.35, 567.60)		546.50 (523.35, 578.35)	0.773
TST [M, (P25, P75)], min		365.00 (313.00, 457.00)		380.75 (305.00, 427.50)		385.50 (329.10, 443.25)	0.483
SE [M, (P25, P75)], %		69.90 (55.60, 81.70)		68.55 (56.15, 78.95)		71.80 (60.60, 82.55)	0.477
SL [M, (P25, P75)], min		13.50 (6.50, 32.50)		23.75 (12.00, 50.85)		23.25 (7.60, 42.00)	0.133
WASO [M, (P25, P75)], min		102.00 (57.00, 154.00)		101.00 (77.50, 154.60)		95.00 (50.85, 154.00)	0.559
N1 [M, (P25, P75)], %TST	38	9.00 (5.80, 14.80)	55	8.75 (5.10, 15.10)	52	9.50 (6.40, 13.80)	0.919
N2 [M, (P25, P75)], %TST		49.20 (37.10, 56.60)		50.60 (38.90, 60.60)		52.20 (45.00, 58.75)	0.612
N3 [M, (P25, P75)], %TST		21.90 (10.50, 34.60)		21.95 (15.30, 31.75)		18.20 (12.65, 27.20)	0.305
R [M, (P25, P75)], %TST		14.70 (12.70, 22.60)		13.40 (9.05, 19.00)		17.80 (14.25, 21.40)	0.011^c^
AHI [M, (P25, P75)], /hr		6.20 (2.30, 17.50)		2.50 (0.25, 8.50)		6.15 (1.75, 19.45)	0.054
PLMSI [M, (P25,P75)], /hr		0 (0, 49.30)		0 (0, 46.00)		0 (0, 59.10)	0.859

*N, number; SD, standard deviation; M, median; P25, 25% percentile values; P75, 75% percentile values*.

a *p-values significant differences were found between PD+RBD and PD-RBD groups*.

b *p-values significant differences were found between PD+RBD and iRBD groups*.

c *p-values Significant differences were found between PD-RBD and iRBD groups*.

### Biomarker Data

**Figure 1:**
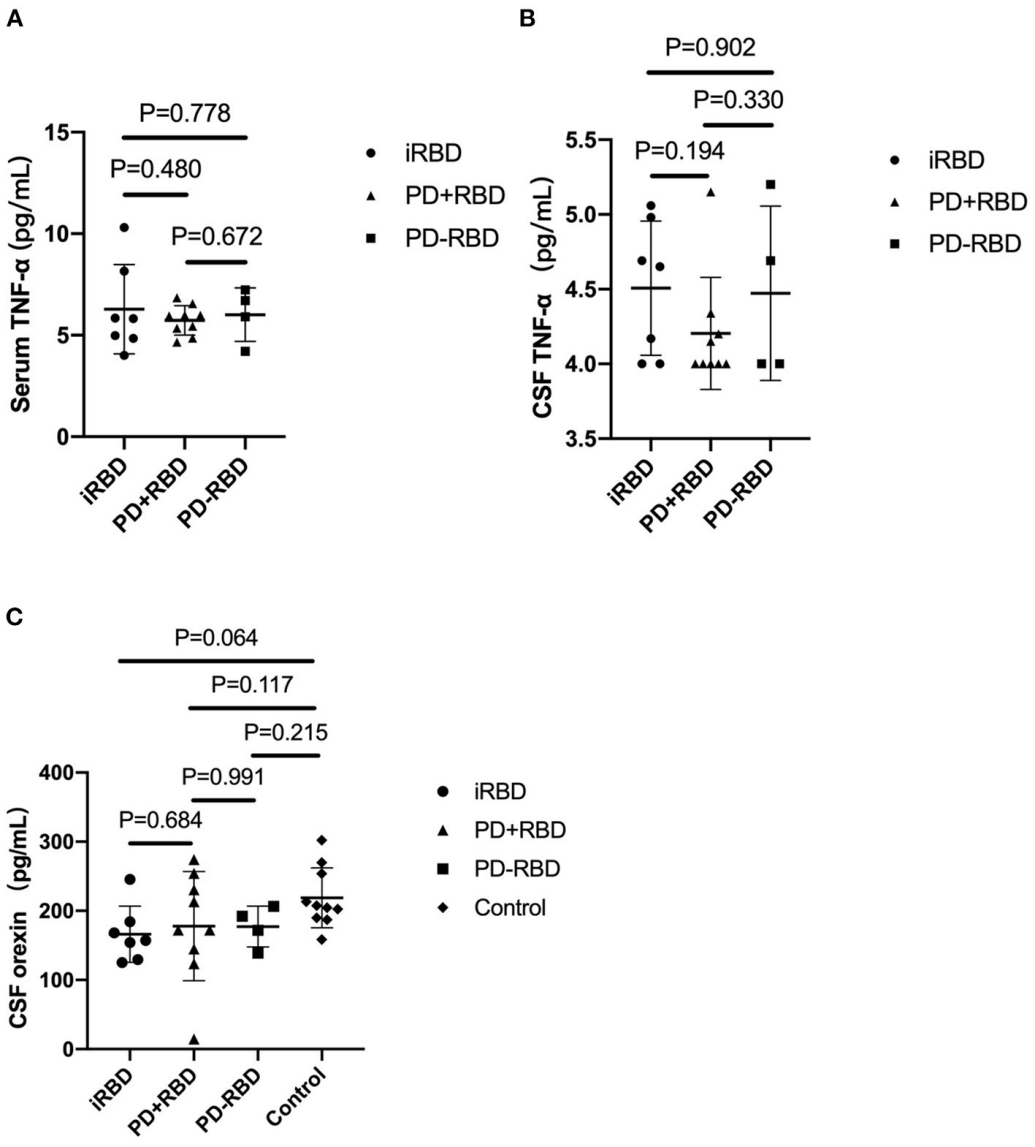
The levels of TNF-α in serum and cerebrospinal fluid (CSF) in PD patients with RBD, PD patients without RBD, and isolated RBD patients **(A,B)**. The levels of orexin in CSF in the four groups **(C)**. Bars represent the mean values, and T bars indicate the standard deviations.

In this study, no significant difference was observed in the serum TNF-α levels among the three groups (5.73 ± 0.73 vs. 6.01 ± 1.31 vs. 4.51 ± 0.45 pg/ml, *P* = 0.773). Also, no significant difference was observed in the CSF TNF-α levels between the three groups (4.20 ± 0.37 vs. 4.47 ± 0.58 vs. 6.28 ± 2.20 pg/ml, *P* = 0.368). CSF orexin levels were abnormal (<200 pg/ml) in all PD+RBD, PD-RBD and iRBD patients (177.69 ± 46.04, 177.31 ± 29.40, 166.23 ± 40.62 pg/ml). Yet the orexin levels of the three groups were not significantly lower than the control group. Furthermore, there was no significant correlation between the levels of TNF-α and orexin (*P* = 0.647) (see Table 2, Figure 1).

**Table 2:** Biomarkers including tumor necrosis factor-alpha (TNF-α) in serum and cerebrospinal fluid (CSF), orexin in cerebrospinal fluid in the three groups.

	**PD+RBD (*n* = 9)**	**PD–RBD (*n* = 4)**	**iRBD (*n* = 7)**	**Control (*n* = 10)**	***P-*value**
Serum TNF-α (mean ± SD), pg/mL	5.73 ± 0.73	6.01 ± 1.31	4.51 ± 0.45	NA	0.773
CSF TNF-α (mean ± SD), pg/mL	4.20 ± 0.37	4.47 ± 0.58	6.28 ± 2.20	NA	0.368
CSF orexin (mean ± SD), pg/mL	177.69 ± 46.04	177.31 ± 29.40	166.23 ± 40.62	218.83 ± 43.36	0.224

## Discussion

This study explored biomarkers in serum and CSF in iRBD and Parkinson's disease patients. Also, we attempt to untangle the relationship between inflammatory marker TNF-α and the orexinergic system. We speculated that TNF-α may not induce the onset and progression of neurodegenerative diseases by acting on the orexin system.

Evidence suggests that CSF and blood biomarkers closely reflecting the pathophysiology of Parkinson's disease, such as major intermediary filament of astrocytes glial fibrillary acidic protein (GFAP) (16), a marker of astroglial activation YKL-40 (17) and proinflammatory cytokines tumor necrosis factor-alpha, IL-6, and IL-12 (12), may have diagnostic and prognostic value.

Inflammation, specifically glial activation, has been linked to the progressive degenerative process in Parkinson's disease. Increased level of TNF-α in CSF from PD patients has been observed in previous studies (18, 19). Furthermore, as with the prodromal phenotype of Parkinson's disease, the neurodegenerative process of iRBD is mediated by TNF-α similarly (13), suggesting that a-synuclein pathologies have already existed in iRBD and play a role in the activation of microglia. However, it is still unclear how it affects the onset and progression of the disease.

Interestingly, TNF-α was found to downregulate orexin levels and exhibit increased REM sleep electromyographic activity in a previously published animal study *via* a protein degradation mechanism (14). As we all know, orexin deficiency is a common feature of narcolepsy type one patients (20). It is unknown whether orexin has any effect on neurodegenerative disease. According to Luppi et al., orexin is indirectly involved in spinal motor neuron innervation in the lower part of the dorsolateral tegmental nucleus (SLD) *via* fiber projections to the pontine tegmental nucleus, and orexin deficiency can cause decreased neuronal excitability in SLD, resulting in muscle atonia in REM sleep (9). This study sheds light on clinical research. However, the results of current studies on the level of orexins in CSF from Parkinson's disease and iRBD patients vary. Several studies have found that the number of orexin neurons in the post-mortem hypothalamus and the level of orexin in ventricular CSF are significantly lower than in controls (21–23). Despite the fact that the differences were not statistically significant, our study found that orexin levels in the three groups decreased when compared to controls. Orexin plays a neuroprotective role in Parkinson's disease *via* a variety of mechanisms, including maintaining the firing of nigral dopamine neurons (24). We believe that the lower levels of orexin found in our study indicate that the orexinergic system dysfunction plays a role in the pathogenesis of Parkinson's disease. Furthermore, functional abnormalities may have occurred during the prodromal stage of Parkinson's disease. However, orexin levels may have no relation to disease progression or phenotype. Another study suggests that high levels of orexin in Parkinson's disease are linked to the loss of REM muscle atonia (25). According to the study, orexin enhances muscle activity *via* both direct effects on spinal motor neurons and indirect effects on locus coeruleus neurons. Furthermore, some studies show no significant reduction in orexin levels in CSF from Parkinson's disease and iRBD patients (26, 27). The difference in CSF orexin levels between their results and our study could be explained by the CSF collection method. Another possible explanation for these disparities is that the patients in the studies were sampled differently. TNF-α levels in serum and CSF of PD patients with RBD were not significantly different from those of other groups in this study, implying that TNF-α may not have a significant effect on the orexinergic system.

Although there was no statistically significant difference, the UPDRS-III score in the PD+RBD group was higher than the PD-RBD group, which is consistent with previous research that PD patients with RBD have more severe motor impairment (28). Except for the fact that the RBDQ-HK score of the PD+RBD and iRBD group was higher than that of the PD-RBD group, we found no significant difference among the three groups in sleep scale scores. Despite the lack of a significant difference, the mean value of MoCA in the PD+RBD group was lower than PD-RBD group. Many studies have found that PD patients with RBD have a higher risk of cognitive impairment (29). The link between RBD and cognitive dysfunction could be due to the brainstem nuclei involved in RBD mediating cognition (30). The study found no more depression in the PD+RBD group than in the other two groups, which is consistent with previous research (31). Furthermore, our study discovered that PD patients with RBD were more anxious than iRBD patients, which may result from comorbidity. Furthermore, a study indicates that dysfunctions in the raphe nucleus and the locus ceruleus cause anxiety in Parkinson's disease patients (32). It has also been reported that PD patients with RBD are more likely to experience anxiety and depression, and it is believed that the presence of RBD causes anxiety and depression in PD patients, which can further affect the sleep quality of patients (33). Sleep architecture did not differ significantly among the three groups, except for a higher proportion of REM sleep in iRBD patients compared to the PD-RBD group.

There are several limitations to this study. First, in patients who do not complain of abnormal behaviors in their sleep, the object of inquiry about the PSQI and RBDQ-HK should be the family members or caregivers of the patient rather than the patient himself. The resulting scale scores more accurately reflect the patients' actual RBD severity. Second, this study didn't further divided patients with or without therapeutic medication into subgroups. After the symptomatic improvement, the results of scales such as UPDRS, RBDQ-HK, HAMA may be different from those obtained prior to medication, and fail to reflect the actual situation, causing bias in the results. Third, PD patients with RBD were not further classified as to whether RBD appeared before or after motor symptoms of PD. We wonder if the time node at which RBD first appears may be related to the severity of neuroinflammatory reactions in the central and peripheral nervous system. Finally, the sample size of this study is small, and more patients should be recruited in the study in the future.

In conclusion, our study investigated biomarkers of phenoconversion in Parkinson's disease and iRBD, as well as the relationship between inflammatory factor TNF-α and orexinergic system in clinical aspect, which might be useful for risk stratification of disease conversion. In the future, the effects of TNF-α and orexin on Parkinson's disease should be studied and discussed in a larger sample size.

## Data Availability Statement

The raw data supporting the conclusions of this article will be made available by the authors, without undue reservation.

## Ethics Statement

The studies involving human participants were reviewed and approved by Xuanwu Hospital, Capital Medical University Institutional Review Board. The patients/participants provided their written informed consent to participate in this study.

## Author Contributions

YY, YZ, YC, YH, ZH, JM, NL, and SZ: conceived and designed the experiments. YY, YZ, YC, YH, ZH, and JM: performed the experiments. YY, YZ, YC, and YH: analyzed the data. YY, YZ, YC, NL, and SZ: wrote the paper. All authors read and approved the content.

## Funding

This work was supported by grants from the National Natural Science Foundation of China (Grant Numbers: 81571294 and 2017YFC0909102) and the Foundation of Chinese Sleep Research Society Hansoh Project.

## Conflict of Interest

The authors declare that the research was conducted in the absence of any commercial or financial relationships that could be construed as a potential conflict of interest.

## Publisher's Note

All claims expressed in this article are solely those of the authors and do not necessarily represent those of their affiliated organizations, or those of the publisher, the editors and the reviewers. Any product that may be evaluated in this article, or claim that may be made by its manufacturer, is not guaranteed or endorsed by the publisher.
